# Lycopene and bone: an in vitro investigation and a pilot prospective clinical study

**DOI:** 10.1186/s12967-020-02238-7

**Published:** 2020-01-29

**Authors:** Cristina Russo, Yvelise Ferro, Samantha Maurotti, Maria Antonietta Salvati, Elisa Mazza, Roberta Pujia, Rosa Terracciano, Giuseppina Maggisano, Rosario Mare, Sandro Giannini, Stefano Romeo, Arturo Pujia, Tiziana Montalcini

**Affiliations:** 1 Department of Clinical and Experimental Medicine, Nutrition Unit, University Magna Grecia, 88100 Catanzaro, Italy; 2 Department of Health Science, University Magna Graecia, 88100 Catanzaro, Italy; 3 Department of Medical and Surgical Science, University Magna Graecia, 88100 Catanzaro, Italy; 4 Department of Medicine, Clinica Medica 1, University of Padova and Regional Centre for Osteoporosis, Padua, Italy; 5 Department of Molecular and Clinical Medicine, Sahlgrenska Center for Cardiovascular and Metabolic Research, University of Gothenburg, 42246 Göteborg, Sweden

**Keywords:** Nutraceutical, Lycopene, Osteoporosis, Bone metabolism, Bone mineral density

## Abstract

**Background:**

There are several effective therapies for osteoporosis but these agents might cause serious adverse events. Lycopene intake could prevent bone loss, however studies on its effects on bone are scarce. Our aim was to investigate the effects of lycopene on osteoblast cells as well as bone mineral density and bone turnover markers in postmenopausal women.

**Methods:**

We investigated the effect of lycopene on the Wnt/β-catenin and ERK 1/2 pathways, RUNX2, alkaline phosphatase, RANKL and COL1A of Saos-2. We also carried out a pilot controlled clinical study to verify the feasibility of an approach for bone loss prevention through the intake of a lycopene-rich tomato sauce in 39 postmenopausal women.

**Results:**

Lycopene 10 µM resulted in higher β-catenin and phERK1/2 protein *Vs* the vehicle (p = 0.04 and p = 0.006). RUNX2 and COL1A mRNA was induced by both 5 and 10 µM doses (p = 0.03; p = 0.03 and p = 0.03; p = 0.05) while RANKL mRNA was reduced (p < 0.05). A significant bone density loss was not detected in women taking the tomato sauce while the control group had bone loss (p = 0.002). Tomato sauce intake resulted in a greater bone alkaline phosphatase reduction than the control (18% vs 8.5%, p = 0.03).

**Conclusions:**

Lycopene activates the WNT/β-catenin and ERK1/2 pathways, upregulates RUNX2, alkaline phosphatase, COL1A and downregulates RANKL Saos-2. These processes contributed to prevent bone loss in postmenopausal women.

## Introduction

Osteoporosis (OP) is a chronic metabolic disease characterized by a low bone mineral density (BMD) and a deterioration in the microarchitecture of the bone which leads to an increased bone fragility and, consequently, fracture [1]. OP is the most common chronic metabolic bone disease affecting hundreds of millions of people worldwide [2]. Despite the current pharmacological agents used to treat OP which are effective in increasing BMD and lowering the risk of fractures, currently less than 20% of patients with a fragility fracture receive therapy to reduce the high risk of fracture [3]. In Europe, treatment uptake for osteoporosis has fallen in recent years, and approximately, 60% of women at high risk do not receive therapy [3].

This decline has, at least in part, been attributable to concerns about the safety of the anti-osteoporotic agents [3]. Irritation of the upper gastrointestinal mucosa, atypical fractures and osteonecrosis of the jaw have been reported with antiresorptive agents, venous thromboembolism has been associated with hormone replacement therapy and hypersensitivity drug reactions and cancer are linked to bone-forming agents [4–7].

New therapeutic strategies devoid of any serious adverse effects are therefore needed to safely treat osteoporotic patients. Several lines of evidence show that nutraceutical compounds can prevent bone loss, which has recently led to their increased prophylactic use, especially in postmenopausal women [8, 9].

Among these, carotenoids play an important role in the regulation of bone metabolism [10]. Carotenoids are mentioned mostly in relation to their antioxidant properties [11]. The conjugated double-bond chain determines their biological functions which includes a scavenger activity on free radicals [12]. A European investigation, demonstrated that a higher dietary intake of carotenoids improves BMD and reduces the osteoporotic fracture risk [10].

Among carotenoids, lycopene is reported to have one of the highest antioxidant capacities [13]. It has been reported that dietary lycopene as well as its supplementation, could be of benefit against the development of numerous chronic diseases including cancer and heart diseases [14, 15]. However, there is a scarcity of in vitro studies investigating the effects of lycopene on bone cells. Moreover, clinical studies investigating the role of lycopene-rich foods in preventing bone loss are lacking.

Considering the abundance of lycopene in the human food supply along with its superior antioxidant function, and the potential adverse events as well as the high cost of the pharmacological agents to treat osteoporosis, further studies are justified to establish the role of lycopene in bone loss protection.

In our study we thus investigated the direct effects of lycopene on osteoblast function. As suggested by previous studies on other areas of health [16–18], here we examined its ability in modulating Wnt/β-catenin and ERK 1/2 signaling in osteoblast-like cells, as well as its effects on differentiation and osteoclastogenesis. We also carried out a pilot prospective clinical study to verify the feasibility of a new therapeutic approach for bone loss prevention through a lycopene-rich tomato sauce intake in a group of postmenopausal women.

## Materials and methods

### Cell culture

The human osteoblast-like cell line Saos-2 was obtained from American Type Culture Collection ATCC (Italy Office, via Venezia 23, 20,099 Sesto San Giovanni, Milan, Italy). The cells were maintained in McCoy’s 5A (Gibco, Carlsbad, CA, USA) supplemented with 15% fetal bovine serum (Gibco, Carlsbad, CA, USA) and 1% penicillin streptomycin (PAA, Linz, Austria), at 37 °C in 5% CO_2_, then harvested by trypsinization and subcultured twice weekly. In all the experiments Saos-2 cells were incubated with dexamethasone 10 nM (Sigma Aldrich, St. Louis, MO, USA) to obtain a more differentiated cell line.

### Cell proliferation

For the evaluation of cell proliferation, Saos-2 cells were seeded at a density of 200,000 cells/well in 6-well dishes. Cells were incubated with lycopene ≥ 98%, from tomato (Sigma Aldrich, St. Louis MO, USA), 5 and 10 µM or vehicle Tetrahydrofuran, THF (Sigma Aldrich, St. Louis MO, USA) in serum free medium for 24 h.

### Western blotting

Saos-2 cells were seeded at a density of 200,000 cells/well in 6-well dishes, and 500,000 cells/well in 100 mm culture dishes. Cells were grown in serum-free medium and incubated with lycopene 5 and 10 µM or vehicle (THF) for 10 min or 24 h.

Cells were lysed in Mammalian Protein Extraction Reagent (M-PER) (Pierce, Thermo Fisher Scientific). Western blot analysis was performed according to standard procedures. The following antibodies were used: rabbit anti β-catenin (19,807), rabbit anti-Runt-related transcription factor 2 (RUNX2) (12,556), rabbit anti-p Extracellular Signal-regulated Kinase (ERK)1/2 (9101) and mouse anti- β-actin (3700), from Cell Signaling Technology (Beverly, MA, USA); rabbit anti-type IA collagen (COL1A) (HPA011795) from Sigma Aldrich (St. Louis, MO, USA); mouse anti-receptor activator of nuclear factor κ-B ligand (RANKL) (sc-377,079) from Santa Cruz Biotechnology (USA); rabbit anti-OPG and mouse anti-Alkaline phosphatase (ALP), from Abcam (Cambridge).

### Real time-PCR

Saos-2 cells were seeded at a density of 1000,000 cells/well in 100 mm culture dishes. Cells were grown in serum-free medium and incubated with lycopene 5 and 10 µM or vehicle (THF) for 24 h. Total RNA from cells were extracted with Trizol reagent (Life technologies, UK) according to manufacturer’s instructions. cDNA was synthesized from 1 µg total RNA, using High-Capacity cDNA Reverse Transcription Kit (Applied Biosystems, Foster City, CA, USA). mRNA expression of RANKL, osteoprotegerin, RUNX2, COL1A, β-CATENIN and β-ACTIN were quantified by real time-PCR using SYBR^®^ Green dye (SYBR^®^ Green PCR Master Mix, Applied Biosystems, Foster City, CA, USA) (see Additional file 1: Table S1).

### ALP Activity

Saos-2 cells were seeded at a density of 200,000 cells/well in 6-well dishes. Cells were incubated with lycopene 5 and 10 µM or vehicle (THF) in serum free medium for 24 h. Cells were lysed with Mammalian Protein Extraction Reagent (M-PER) (Pierce, Thermo Fisher Scientific). Protein concentration was determined using Bradford assay, and ALP activity was determined by p-nitrophenyl phosphate (pNPP) colorimetric method (WAKO Chemicals USA, Richmond, VA, USA).

### Human study design

A convenience sample of 39 consecutive postmenopausal patients attending the outpatient clinic of the “Mater Domini” Azienda University Hospital in Catanzaro, Italy, were enrolled for this study to receive 150 ml/day of a tomato sauce (provided by C.G. Food, SRL, Soverato, Italy) for 3 months (enrolment period between May 21, 2015 and July 28, 2015). A sample of 39 age-, BMI-matched postmenopausal women not taking this tomato sauce served as control group. For this pilot study, the tomato sauce was the same of the study listed on the ISRCTN registry (study ID ISRCTN13244115; requested patent). Written Informed consent was obtained from all individual participants included in the study. The protocol was approved by the local ethical committee at the “Mater Domini” Azienda University Hospital (117/2015/CE, approved May 14, 2015). The investigation conforms to the principles outlined in the declaration of Helsinki.

In this study we included postmenopausal women in which postmenopausal status was defined as the presence of a serum follicle-stimulating hormone (FSH) level of over 40 IU/l (if available) or no natural menses for at least 1 year. The exclusion criteria were as follows: use of anti‐osteoporotic agents, hypercalcemia, cancer and all the clinical conditions or medications affecting bone metabolism (such as kidney, liver, thyroid and rheumatic diseases, malabsorption syndromes, haematological diseases, use of glucocorticoids, aromatase inhibitors, thyroid hormone replacement, and antiepileptics). If participants took calcium and/or vitamin D supplementation at the time of enrolment, they continued on the same dose throughout the duration of the study.

Patients’ age, BMI (kg/m^2^; dry weight in meters squared), waist and hip circumferences (WC and HC), systemic blood pressure, transaminases, glucose, creatinine, high sensitivity C-reactive protein (CRP), bone-specific alkaline phosphatase (BAP), cross-linked C-terminal telopeptide of type I collagen (s-CTx) in the serum, as well as the presence of diabetes mellitus, renal disease, hyperlipidemia, hypertension, were recorded at baseline.

Quantitative ultrasound (QUS) was used to measure the speed of sound (metres per second) and broadband ultrasound attenuation (BUA) (decibels per megaHertz) of the heel (Sahara^TM^ Clinical Bone Sonometer, Technologic Srl—Hologic, Italia). In cases of a previous fracture within the lower extremity, only the opposite calcaneus was measured. T-score was derived from the value of BUA and expressed as the number of SDs from the mean value of a control gender-matched population [19]. The T-scores are reported as the number of standard deviations below the young adult mean (normal, > −1; osteopaenia, − 1 to − 2.49; osteoporosis, ≤ −2.5) [19]. The coefficient of variation (CV %) was 2% for BUA. Calcaneus BMD, T-score, BAP and s-CTx were measured at baseline and 12 weeks.

### Clinical outcomes

Primary outcomes were: 1. Change in BMD, which was assessed by QUS on individuals’ right calcaneus [20]; 2. Change in key bone turnover markers such as serum BAP and s-CTx.

### Participants biochemical evaluation and lycopene content analysis

Venous blood was collected after fasting overnight into vacutainer tubes (Becton & Dickinson, Plymouth, England) and centrifuged within 4 h. Serum glucose, total cholesterol, high density lipoprotein (HDL)-cholesterol, triglycerides, creatinine, CRP and transaminases were measured by chemiluminescent immunoassay on COBAS 8000 (Roche, Switzerland), according to the manufacturer’s instructions. LDL- C level was calculated by the Friedewald formula.

BAP and s-CTx were measured by chemiluminescent immunoassay on Liaison^®^ XL (DiaSorin, Italy), according to the manufacturer’s instructions. Quality control was assessed daily for all determinations.

Participants in the intervention group received a tomato sauce from tomatoes ripened on-the-vine, which are particularly mature and rich in lycopene. Lycopene concentration in the tomato sauce was assessed by high-performance liquid chromatography (UltiMate 3000 Standard HPLC System, Thermo Fisher Scientific, Italy) [21]. Lycopene (all *trans*-isomeric forms) content was 1.3 mg/50 g of tomato sauce.

### Statistics

Data are reported as mean ± standard deviation.

In relation to the in vitro study, data resulted from a mean of at least three independent experiments, and were analyzed with GraphPad Prism 5.0 software using a two-tailed Student’s *t* test.

For the human study, a Chi square test was performed to analyze the prevalence between groups and an independent unpaired samples t test was used to compare the difference between means. Specifically, we calculated the changes in variables (such as T-score, BMD, BAP. S-CTx) and compared the means of these changes between treatment groups. Changes in the clinical characteristics from baseline to follow-up (within group variation) were calculated using paired Student’s t test (two tailed). The General Linear Model was used to adjust the BMD and BAP change for potential confounders (such as calcium and vitamin D supplementations).

We used a patient interviews to assess adherence to the treatment. All comparisons were performed using SPSS 22.0 for Windows (IBM Corporation, New York, NY, USA). In both the studies significant differences were assumed to be present at p< 0.05 (two-tailed).

## Results

### Lycopene does not affect osteoblast proliferation in vitro

To test the hypothesis that lycopene increases osteoblast proliferation, Saos-2 cells were incubated with lycopene 5 and 10 µM or vehicle for 24 h. Then proliferation was determined by counting the cells number. There were no differences between all doses of lycopene (Additional file 1: Figure S1a).

### Lycopene increases β-catenin protein expression levels, but does not influence mRNA levels on Saos-2 cells

**Fig. 1:**
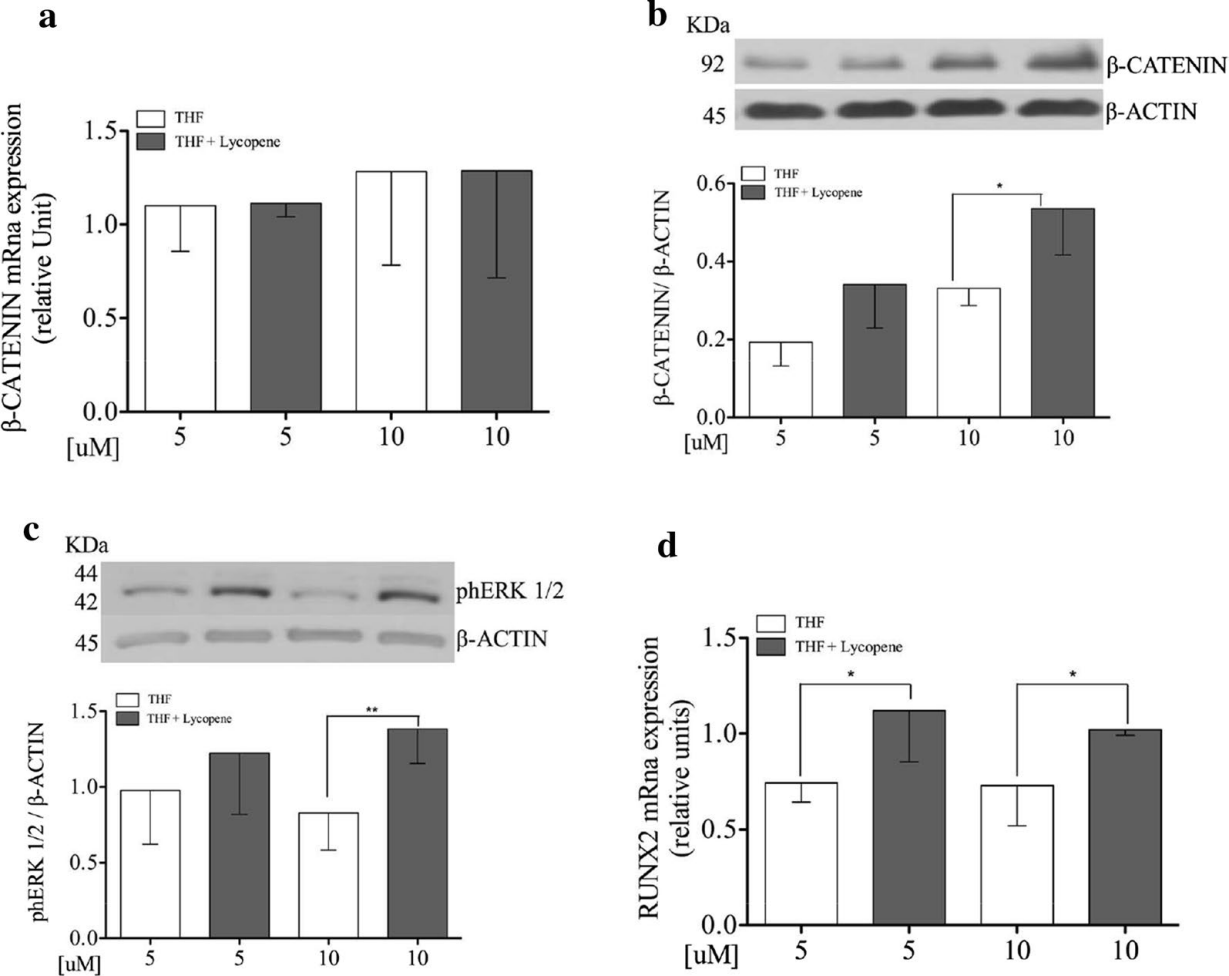
Lycopene increases β-catenin, phErk and RUNX2 protein expression levels on Saos-2 cells. Semi-confluent cultures of human osteoblast-like cells (Saos-2) were incubated with lycopene 5 and 10 μM or vehicle THF in serum free medium for 24 h. mRNA expression levels of β-catenin (**a**) and RUNX2 (**d**) were measured by RT-Pcr. Data were analyzed using the 2-ΔΔCq method and normalized to β-actin. β-catenin and phERK1/2 protein expression is showed in panel **b** and **c**. **b** β-catenin and β-actin proteins were analyzed by Western blotting with specific antibodie. **c** PhERK1/2 and β-actin proteins were analyzed by Western blotting with specific antibodie. Data are represented as mean ± SD. Statistical analysis: Student’s t-test vs THF 10 μM *p < 0.05. *THF* tetrahydrofuran, *RUNX2* Runt-related transcription factor 2

To test the effect of lycopene on β-catenin pathway, Saos-2 cells were incubated with lycopene for 24 h at different doses. As shown in Fig. 1a, lycopene does not affect the mRNA expression levels of β-catenin at all doses. However, exposure of Saos-2 cells to lycopene 10 µM, resulted in higher active β-catenin protein expression than the vehicle 10 µM (p = 0.04; Fig. 1b).

### Lycopene induces phERK1/2 protein expression level on Saos-2

Saos-2 cells were incubated with lycopene 5 and 10 µM or vehicle for 10 min. Lycopene incubation at the dose of 10 µM resulted in higher phERK1/2 protein expression levels than the respective vehicle (p = 0.006; Fig. 1c).

### Lycopene increases mRNA expression levels of RUNX2 as well as of the COL1A, and decreases RANKL mRNA expression levels on Saos-2

**Fig. 2:**
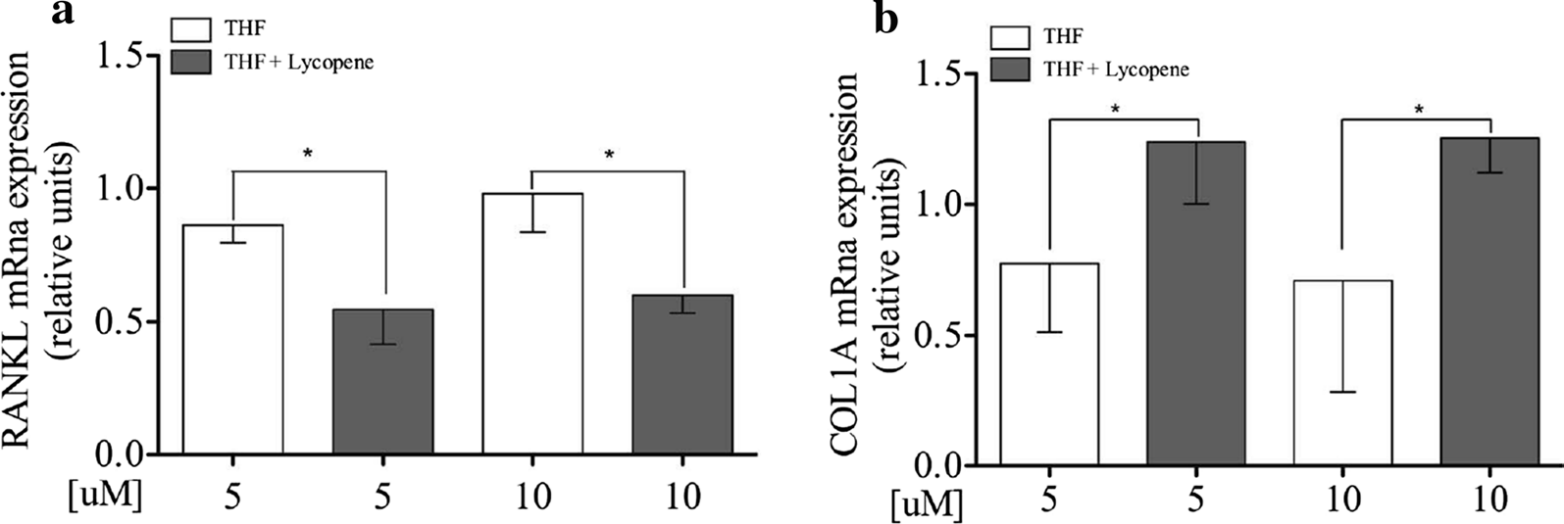
Lycopene decreases RANKL mRNA expression levels and increases COL1A mRNA levels on Saos-2 cells Semi-confluent cultures of human osteoblast-like cells (Saos-2) were incubated with lycopene 5 and 10 µM or vehicle THF in serum free medium for 24 h. mRNA expression levels of RANKL (**a**) and COL1A (**b**) were measured by RT-Pcr. Data were analyzed using the 2-ΔΔCq method and normalized to β-actin. Data are represented as mean ± SD. Statistical analysis: Student’s t-test vs THF 5 and 10 µM respectively, *p < 0.05. *THF* tetrahydrofuran, *RANKL* receptor activator of nuclear factor-kB ligand, *COL1A* type 1A collagen

Saos-2 cells were exposed to lycopene at 5 and 10 µM doses or vehicle at the same concentration. RUNX2 mRNA expression level was significantly induced by both 5 and 10 µM doses in comparison to their respective vehicles (p = 0.03 and p = 0.03, respectively; Fig. 1d). The incubation of Saos-2 cells with both 5 and 10 µM doses resulted in lower RANKL mRNA expression levels than their respective vehicles (p = 0.0207 and p = 0.0145, respectively; Fig. 2a).

As shown in Fig. 2b, exposure to lycopene 5 and 10 µM resulted in higher COL1A mRNA levels in comparison with their respective vehicles (p = 0.0379 and p = 0.050, respectively). Lycopene did not affect Osteoprotegerin mRNA expression (Additional file 1: Figure S1b).

### Lycopene induces higher mRNA and protein expression of ALP and increases its enzymatic activity on Saos-2 cells

**Fig. 3:**
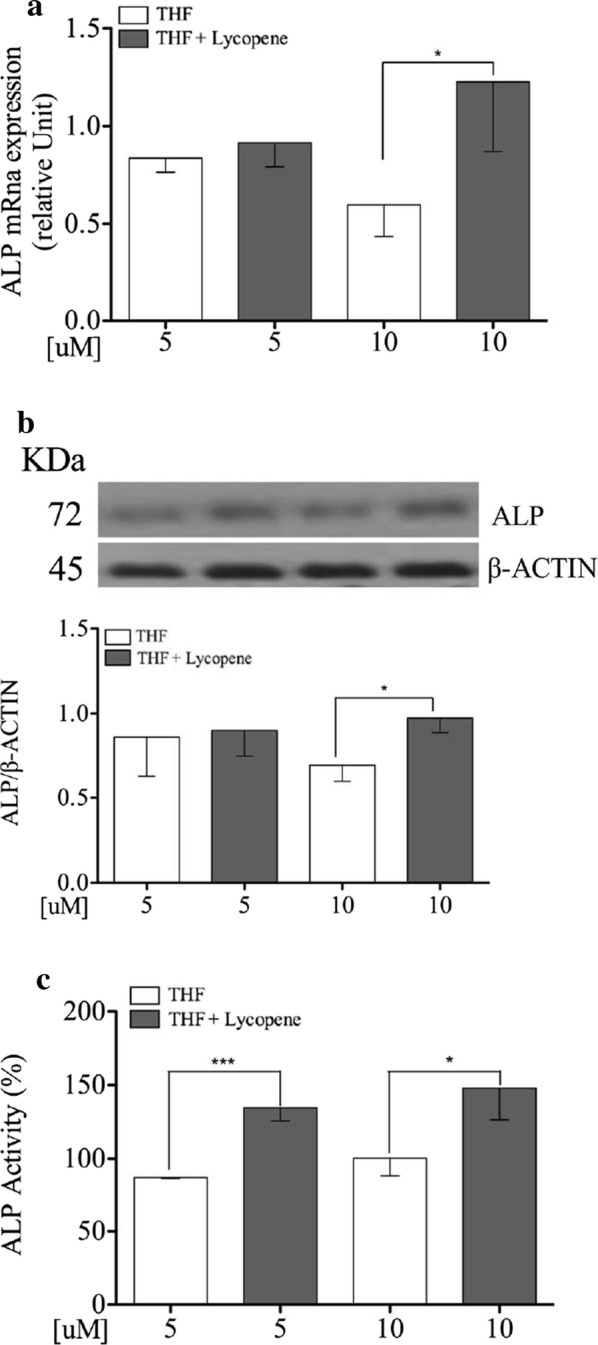
Lycopene increases mRNA, protein and activity of Alkaline Phosphatase on Saos-2 cells Semi-confluent cultures of human osteoblast-like cells (Saos-2) were incubated with lycopene 5 and 10 µM or vehicle THF in serum free medium for 24 h. **a** mRNA expression levels of alkaline phosphatase were measured by RT-Pcr. Data were analyzed using the 2-ΔΔCq method and normalized to β-actin. **b** Cell proteins were analyzed by Western blotting with antibodies specific to alkaline phosphatase and β-actin. **c** ALP activity was measured by pNPP method. Data are represented as mean ± SD. Statistical analysis: Student’s t-test vs THF 5 and 10 µM respectively, *p < 0.05; ***p < 0.001. *THF* tetrahydrofuran

To test the hypothesis that lycopene acts on bone biomarker ALP, Saos-2 cells were incubated with lycopene 5 and 10 µM or their respective vehicles for 24 h. The exposure to 10 µM dose resulted in higher mRNA and protein ALP expression levels than the vehicle (p = 0.04 and p = 0.01 respectively; Fig. 3a, b). The ALP activity was significantly increased with lycopene 5 and 10 µM in comparison to their vehicles (p < 0.001 and p = 0.02, respectively, Fig. 3c).

### Clinical characteristics of participants in the pilot study

The mean age of the enrolled population was 63 ± 7 years. The mean basal BMD was 0.40 ± 0.08 g/cm^2^ and mean T-score was −1.65 ± 0.77 SD (osteopenia). Additional file 1: Table S1 shows the clinical characteristics of participants according to treatment. The groups were comparable for all of the characteristics.

### Clinical characteristics changes at follow-up and outcomes of the study

Changes in the clinical parameters after each treatment period are shown in Table 1.

**Table 1:** Changes of the characteristics of participants at follow-up according to intervention

Variables	Without tomato sauce (n = 39)	With tomato sauce (n = 39)	P-value
BMI (Kg/m^2^)	− 0.31 ± 0.67	− 0.20 ± 0.72	0.49
T-score (SD)	− 0.15 ± 0.3	0.00 ± 0.2	0.05
BMD (g/cm^2^)	− 0.02 ± 0.04	0.00 ± 0.03	0.006
BMD (g/cm^2^)^a^	− 0.028 ± 0.006	0.005 ± 0.0006	0.001
BMD (%)	− 5.3 ± 10	0.08 ± 7	0.009
Glucose (mg/dL)	− 0.74 ± 6	− 3.05 ± 7	0.15
Creatinine (mg/dL)	− 0.02 ± 0.07	0.00 ± 0.08	0.23
CRP(mg/L)	− 0.65 ± 3	0.01 ± 0.6	0.32
S-CTX (ng/mL)	− 0.003 ± 0.13	− 0.001 ± 0.12	0.95
BAP (µg/L)	− 1.95 ± 3	− 3.53 ± 3	0.05
BAP (µg/L)^a^	− 1.79 ± 0.6	− 3.69 ± 0.6	0.04
BAP (%)	− 8.5 ± 21	− 18 ± 17	0.03

*BMI* body mass index, *BMD* bone mineral density, *S-CTx* serum carboxyterminal crosslinked telopeptide of type I collagen, *BAP* bone alkaline phosphatase, *CRP* C-reactive protein

^a^Adjusted for calcium and vitamin D supplementation

The adjusted BMD change was 0.005 ± 0.006 and −0.028 ± 0.006 g/cm^2^ in the tomato sauce and the control group, respectively (p = 0.001) and the adjusted BAP change was −3.69 ± 0.6 and −1.79 ± 0.6 ug/L in the tomato sauce and the control group, respectively (p = 0.040; Table 1). Tomato sauce resulted in a greater BAP reduction than in the control group (18% vs 8.5%, p = 0.03; Table 1). The BMD percent change, after 12 weeks, was 0.08% with Tomato sauce and −5.3% in the control group (p = 0.009, Table 1). No other variables were significantly different between groups at follow-up visit, except, as expected, T-score (Table 1). The participants in the tomato sauce group had a high adherence to the protocol (i.e.; > 80% of the prescribed treatment).

**Fig. 4:**
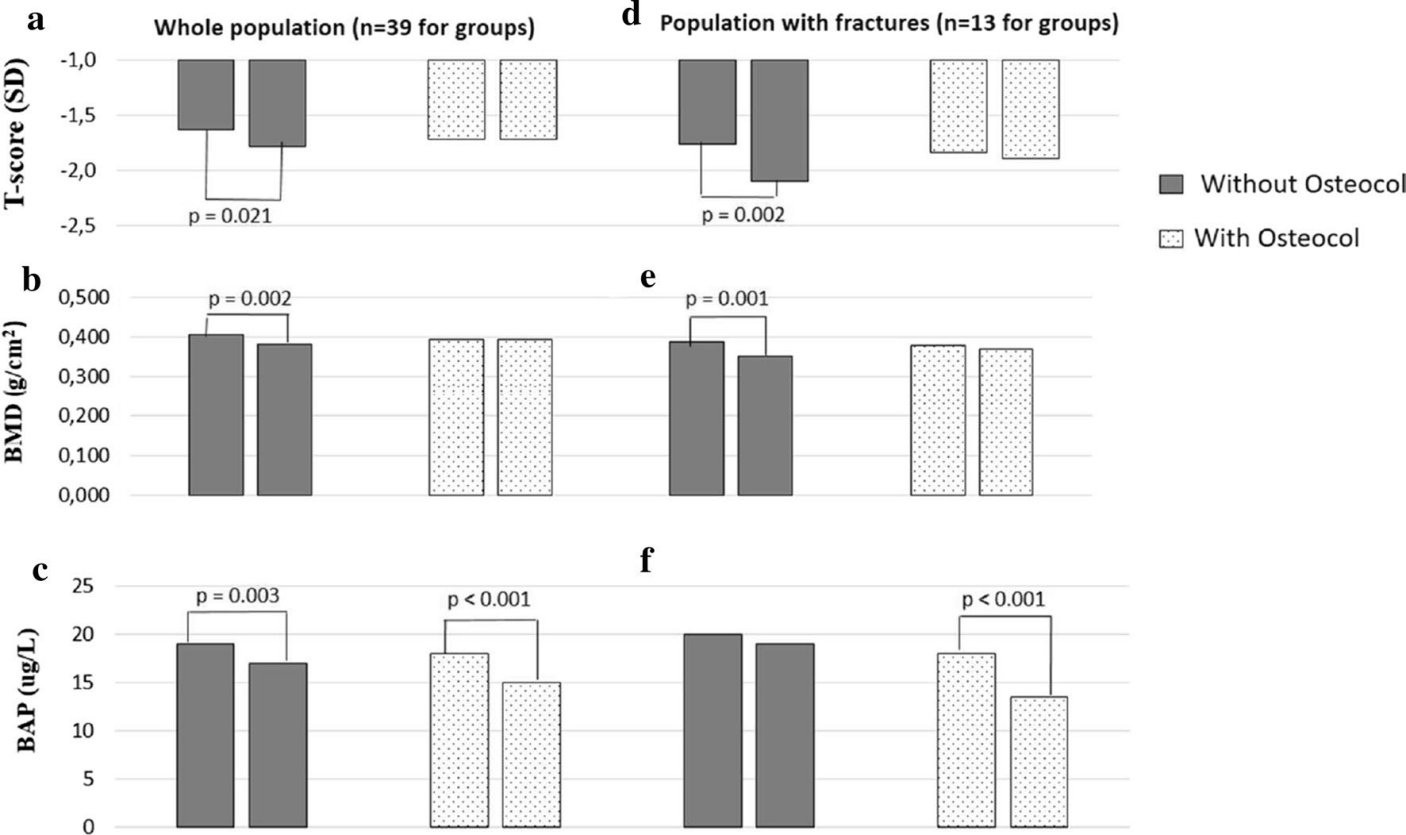
Changes in bone parameters after 12 weeks Graphical representation of the whole population and of the population with fractures after 12 weeks with or without lycopene rich-tomato-souce of the T-Score (**a** and **d** respectively); BMD (**b** and **e** respectively) and BAP (**c** and **f** respectively). *BMD* bone mineral density, *BAP* bone alkaline phosphatase

A significant loss of BMD was not detected in the participants consuming the tomato sauce in comparison to their baseline (0.393 ± 0.07 to 0.393 ± 0.08 g/cm^2^; p = 0.97, Fig. 4b), while women who did not consume the tomato sauce experienced BMD reduction compared to the their baseline (0.406 ± 0.09 to 0.382 ± 0.08 g/cm^2^; p = 0.002; Fig. 4b). T-score had a similar reduction in the two groups (Fig. 4a). A significant reduction of BAP was detected in both the groups in comparison to the baseline (in the Tomato sauce group, from 18.1 ± 7.0 to 14.6 ± 5.9 ug/L; p < 0.001; in the control group, from 19.2 ± 6.9 to 17.2 ± 6.7 ug/L; p = 0.003; Fig. 4c). A subgroup analysis of patients with fractures highlighted the efficacy of the tomato sauce in preventing BMD reduction and reducing BAP (Fig. 4d–f).

## Discussion

In our study, we examined the in vitro effects of lycopene (≥ 98%, from tomato) on the osteoblast metabolism. We demonstrated that lycopene exposure influences osteoblast-like cell Saos-2 function in several ways:

- It activates two key cellular pathways such as ERK 1/2 and WNT/β-catenin;
- It upregulates RUNX2 mRNA levels;
- Lycopene increases mRNA, protein and activity levels of ALP; while downregulates RANKL;
- It upregulates COL1A mRNA.

The pilot clinical study demonstrated that postmenopausal women that consumed a lycopene-rich tomato sauce daily, from tomatoes ripened on-the-vine at a dose of 150 mg, prevent bone loss in a 3 month period, while those who did not consume the tomato sauce reduced their BMD. In addition, those women who consumed the tomato sauce reduced their BAP to a greater extent than those women who did not consume the tomato sauce.

It is known that carotenoid intake is associated with a lower risk of osteoporosis [10]. However, to date, the effects of lycopene on the bone cell metabolism have scarcely been studied. Since the effects of lycopene on osteoporosis have not been studied previously in human studies, we believe that our results provide new insights.

We found that lycopene did not affect proliferation of Saos-2 cells after 24 h (Additional file 1: Figure S1a). Our finding differs from that of Kim et al. [22] who found a stimulatory effect of lycopene, however they used a microemulsion lycopene preparation at doses that were consistently greater than ours. In a previous study carried out in the clonal osteogenic MC3T3-E1 cell line, lycopene inhibited cell proliferation at a dose of 1 µM and increased alkaline phosphatase activity [23]. We speculate that the inhibitory effect of low lycopene levels is interfered by a secondary stimulatory mechanism that prevails at high lycopene levels. However, the effect of lycopene on osteoblast proliferation needs further clarification.

It has been demonstrated that lycopene exerts anti-tumor properties by the WNT/β-catenin pathway [24, 25]. Since other investigations have suggested that canonical WNT/β-catenin signaling plays a role in controlling the osteoblast differentiation via RUNX2 expression [26, 27], we tested the effect of lycopene on this pathway. In line with these previous findings, we confirmed that lycopene acts by the WNT/β-catenin pathway (Fig. 1a, b). However, in our study lycopene did not affect β-catenin mRNA expression while did affect the protein expression. A poor correlation between protein and mRNA levels may be consistent with the delayed transport of mRNA from the nucleus to cytosol, which could affect cells undergoing long-term dynamic processes such as differentiation [28].

In our study, we found that lycopene activates phERK1/2 protein expression (Fig. 1c), which is known to be involved in differentiation [29]. No previous studies have shown a similar effect on bone cells. It has been reported that retinoids, whose precursors are carotenoids, increase phERK1/2 levels, thus regulating chondrocyte differentiation and proliferation [30, 31]. One study demonstrated that the astaxanthin carotenoid ameliorates the osteogenic differentiation of neural stem cells by activating phERK [32]. Despite scarce evidences, all these previous studies confirm our findings.

In addition, all-trans-retinoic acid has been reported to induce the early osteoblastic transcription factor, RUNX2 expression [33]. Oliveira et al. [34] found an upregulation of RUNX2 in lycopene supplemented ovariectomized rats. In line with these investigations, we found that lycopene, at both doses, increased RUNX2 mRNA levels (Fig. 1d) thus confirming our hypothesis that lycopene influences osteoblast differentiation. RUNX2 is crucial for the commitment of mesenchymal stem cells to the osteoblast lineage [35]. Interestingly, regulation by RUNX2 stimulates cells differentiation at early stages, while it inhibits the process at later stages [35]. Since alendronate promotes osteoblast differentiation [36], our results could have important therapeutic implications.

In our study, we also examined the effect of lycopene on the RANKL mRNA expression on Saos-2 cells. Similar to other carotenoids [37, 38], lycopene downregulates RANKL, and thus could potentially suppress bone resorption. This is the main finding of this study, which has important therapeutic implications.

Another important finding is the stimulatory effect of lycopene on ALP within 24 h.ALP has become the marker of choice when assessing the phenotype or developmental maturity of mineralized tissue cells [39]. It is involved in bone matrix vesicle formation, which ultimately result in the nucleation and propagation of mineral crystals [30].We found that lycopene stimulates this marker at several levels (Fig. 3). Albeit with a delayed effect compared to our findings, other studies have found similar results [22, 23, 33]. Our study suggests the role of lycopene in structuring the matrix vesicles at the site of initial mineral formation [39].

Lastly, we found that lycopene increases COL1A mRNA expression (Fig. 2b). COL1A, the main component of the bone matrix, plays a key role in transferring stress and resisting deformation and fractures [40]. We hypothesize that lycopene may promote bone resistance and repair by influencing collagen biosynthesis.

In the pilot clinical study, we demonstrated the feasibility of a new therapeutic approach to preventing bone loss. We found that a lycopene-rich tomato sauce prevented BMD loss and significantly reduced BAP concentrations by 10% in a 6-week period in postmenopausal women (Table 1 and Fig. 4).

At the beginning of anti-resorptive treatment, bone resorption rapidly decreases [41]. The inhibition of new basic multicellular units (activation frequency) and a decreased differentiation and activity of existing osteoclasts, determine the decrease in the bone turnover markers [41]. These two mechanisms may explain our finding. During the anti-resorptive treatments, short-term decrease in bone formation markers is associated with higher long-term increase in BMD [42]. In a study, by 12 weeks, BAP had decreased significantly in response to both alendronate 10 mg/day and cyclical etidronate (of ~ 10% as in our study) [43]. Thus, we believe that our finding may have important clinical implications.

The change observed in our study in BAP was in the range of those obtained with raloxifene, which is a well-known antiresorptive agent [44]. In addition, a previous investigation demonstrated early changes in calcaneus ultrasound and bone markers, confirming the plausibility of our finding [45]. There is a correlation between the US transmission velocity and the trabecular bone [46].We thus believe that this pilot study represents a necessary first step in exploring novel applications for lycopene rich-foods in future larger scale studies.

The ideal intake of tomatoes or tomato derives is currently unknown. The content of lycopene may decrease according to the storage conditions [47]. The studies on foods in which lycopene is added as an ingredient by the food industries have not yet clarify lycopene nutritional contribution in terms of availability. In this regard, it has been demonstrated a low increase of lycopene in the serum after consumption of some lycopene-enriched formulations [48] and that the process of lycopene absorption is saturable at very low dosing levels [49]. The maintenance of the natural matrix and the addition of a small quantity of fat to the final product may result in a good strategy to achieve a biological response by lycopene rather than adding lycopene in foods [50].

Our pilot study has some limitations. First, study did not include a true control group. Second, due to ethical considerations (unnecessary X-ray exposure) and cost-effective evaluations, we did not perform a dual energy X-ray absorptiometry (DXA) evaluation. In this pilot study, we assessed the feasibility of some key steps (such as recruitment rate, retention levels and eligibility criteria). The focus in the results of this study should be on feasibility, rather than statistical significance, thus, the conclusions drawn from this study should be considered with caution. The presence of other factors and dietary components which may have influenced the results cannot be ruled out.

In our study, lycopene increased ALP expression and activity in vitro, while lycopene-rich tomato sauce decreased BAP in vivo. As previous demonstrated [22, 44, 51], this is not a discrepancy. Moreover, several studies on the bisphosphonate alendronate showed a BAP reduction after therapy and this decrease in BAP was associated with long-term increases in BMD [52].

Other limitations need to be considered. In order to study bone metabolism and the activity of factors involved, various transgenic and knockout rodent models have been proposed [53]. A combination of estrogen deficiency plus a calcium wasting diet in skeletally mature animals or a mouse model of senile osteoporosis should be used [54]. Furthermore, to mimic specific aspects of human osteoporosis, some cellular models provide excellent opportunities. In this regard, oxidative stress have been recognized as crucial players in the pathogenesis of osteoporosis. Unresolved oxidative stress stimulates osteoclast formation and osteocyte apoptosis, and affects osteoblast functions [55]. Unfortunately, in this study we have not performed experiments with the osteoblast pre-exposed to oxidative stress by stimuli such as chemical, physical agents or environmental factors. Of course, in vivo studies should also be performed to confirm our findings. However, there is no models exactly miming the condition of human osteoporosis. We used a Saos-2 cell line, which represent a valuable tool to identify targets and test novel drugs for osteoporosis therapy [56]. Saos-2 cell line exhibit a mature osteoblast phenotype and, under different stimuli, express a panel of factors (such BAP) as highly similar to primary human osteoblastic cells [57]. Independent of the stimuli, the Saos-2 cell line, thus, is a useful model as a source of bone-related molecules.

## Conclusions

Our study shows that lycopene may positively affect osteoblast metabolism by positively influencing osteoblast differentiation and collagen production. In addition, lycopene suppresses RANKL expression, thus indicating a possible role in blocking bone resorption. Its effect on ALP activity also suggests a possible role in the calcification process. Further studies are warranted to investigate the potential relationship between lycopene and bone loss. Clinical studies are especially needed to confirm the potential therapeutic action of lycopene on metabolic bone diseases.

## Supplementary information

**Additional file 1: Table S1.** Real-Time primer sequences**. Table S2.** Baseline demographic and clinical characteristics of participants according to intervention.

## Data Availability

The datasets generated during and/or analysed during the current study are available from the corresponding author on reasonable request.

## Abbreviations

ERK ½: Extracellular Signal-Regulated Kinase 1/2
RUNX2: Runt-related transcription factor 2
ALP: Alkaline phosphatase
RANKL: Receptor activator of nuclear factor κ-B ligand
COL1A: Type 1A Collagen
OP: Osteoporosis
BMD: Bone mineral density
THF: Tetrahydrofuran
pNPP: p-nitrophenyl phosphate
FSH: Follicle-stimulating hormone
BMI: Body mass index
WC: Waist circumference
HC: Hip circumference
BAP: Bone-specific alkaline phosphatase
s-CTx: Cross-linked C-terminal telopeptide of type I collagen
QUS: Quantitative Ultrasound
BUA: Broadband ultrasound attenuation
DXA: Dual energy X-ray absorptiometry
