# Supplementary material for: Lycopene and bone: an in vitro investigation and a pilot prospective clinical study
Source: J Transl Med. 2020 Jan 29;18:43. doi: 10.1186/s12967-020-02238-7 (PMC6990577; doi:10.1186/s12967-020-02238-7)

| ***Additional table 1*. Real-Time primer sequences.** | | |
| --- | --- | --- |
| **Gene** | **Forward** | **Reverse** |
| **RUNX2** | 5’-TTACTTACACCCCGCCAGTC -3’ | 5’-TATGGAGTGCTGCTGGTCTG-3’ |
| **ALP** | 5’-GACCTTGACCCCCACAAT-3’ | 5’-GCTCTACTGCATTCCCCTC-3’ |
| **COL1A** | 5’-CCCCAGCCCACAAAGAGTCTA -3’ | 5’-CTGTACGCAGGTGATTGGTG-3’ |
| **RANKL** | 5’-AGAGCGCAGATGGATCCTAA-3’ | 5’-TTCCTTTTGCACAGCTCCTT-3’ |
| **β- CATENIN** | 5′-ATGGAGCCGGACAGAAAAGC-3′ | 5′CTTGCCACTCAGGGAAGGA-3′ |
| **Osteoprotegerin** | 5’-TGCAGTACGTCAAGCAGGAG-3’ | 5’-GTGTCTTGGTCGCCATTTTT-3’ |
| **β-ACTIN** | 5’-GACTGTGACGAGTTGGCTGA-3’ | 5’-CTGGAGAGGAGCAGAACTGG-3’ |

| *Additional table 2.*Baseline demographic and clinical characteristics of participants according to intervention | | | | |  |
| --- | --- | --- | --- | --- | --- |
| Variables | | Without Tomato sauce  (n=39) | With Tomato sauce  (n=39) | *p-value* |  |
| Age (years) | | 63±7 | 63±7 | 0.77 |  |
| Age at menopause (years) | | 14±7 | 15±7 | 0.83 |  |
| Weight (Kg) | | 65±10 | 65±10 | 0.89 |  |
| BMI (Kg/m^2^) | | 26.8±4 | 27.6±4 | 0.41 |  |
| WC (cm) | | 89±11 | 90±10 | 0.74 |  |
| HC (cm) | | 103±10 | 104±9 | 0.76 |  |
| SBP (mmHg) | | 124±17 | 125±13 | 0.70 |  |
| DBP (mmHg) | | 72±8 | 71±9 | 0.68 |  |
| T-score (SD) | | -1.63±0.76 | -1.67±0.80 | 0.81 |  |
| BMD (g/cm^2^) | | 0.406±0.08 | 0.399±0.08 | 0.69 |  |
| Glucose (mg/dL) | | 90±9 | 93±9 | 0.18 |  |
| Creatinine (mg/dL) | | 0.71±0.1 | 0.72±0.1 | 0.53 |  |
| AST (IU/L) | | 20±5 | 19±3 | 0.19 |  |
| ALT (IU/L) | | 20±11 | 16±5 | 0.04 |  |
| CRP(mg/L)  S-CTX (ng/mL) | | 3.3 ±0.9  0.587±0.22 | 4.3± 3  0.512±0.16 | 0.16  0.09 |  |
| BAP (ug/L) | | 19.2±6.9 | 18.1±7.0 | 0.51 |  |
| *Prevalences* | | | | | |
| Smokers (%) | 18 | | 10 | 0.51 | |
| Hyperlipidemia (%) | 51 | | 41 | 0.49 | |
| Hypertension (%) | 39 | | 46 | 0.64 | |
| DMT2 (%) | 0 | | 3 | 1 | |
| Total fractures (%) | 33 | | 33 | 1 | |
| *Medications* | | | | | |
| Calcium (%) | 5 | | 21 | 0.08 | |
| Vitamin D (%) | 23 | | 33 | 0.45 | |
| *Note.*BMI = body mass index; WC = waist circumference; HC = hip circumference; SBP = systolic blood pressure; DBP = diastolic blood pressure; BMD = bone mineral density; TC = total cholesterol; HDL-C = high density lipoprotein cholesterol; LDL-C = low density lipoprotein cholesterol; TG = triglycerides; AST = aspartate aminotransferase; ALT = alanine aminotransferase; S-CTx = serum carboxyterminal crosslinked telopeptide of type I collagen; BAP = bone alkaline phosphatase; DMT2 = diabetes mellitus type 2. | | | | |  |

Additional figure 1


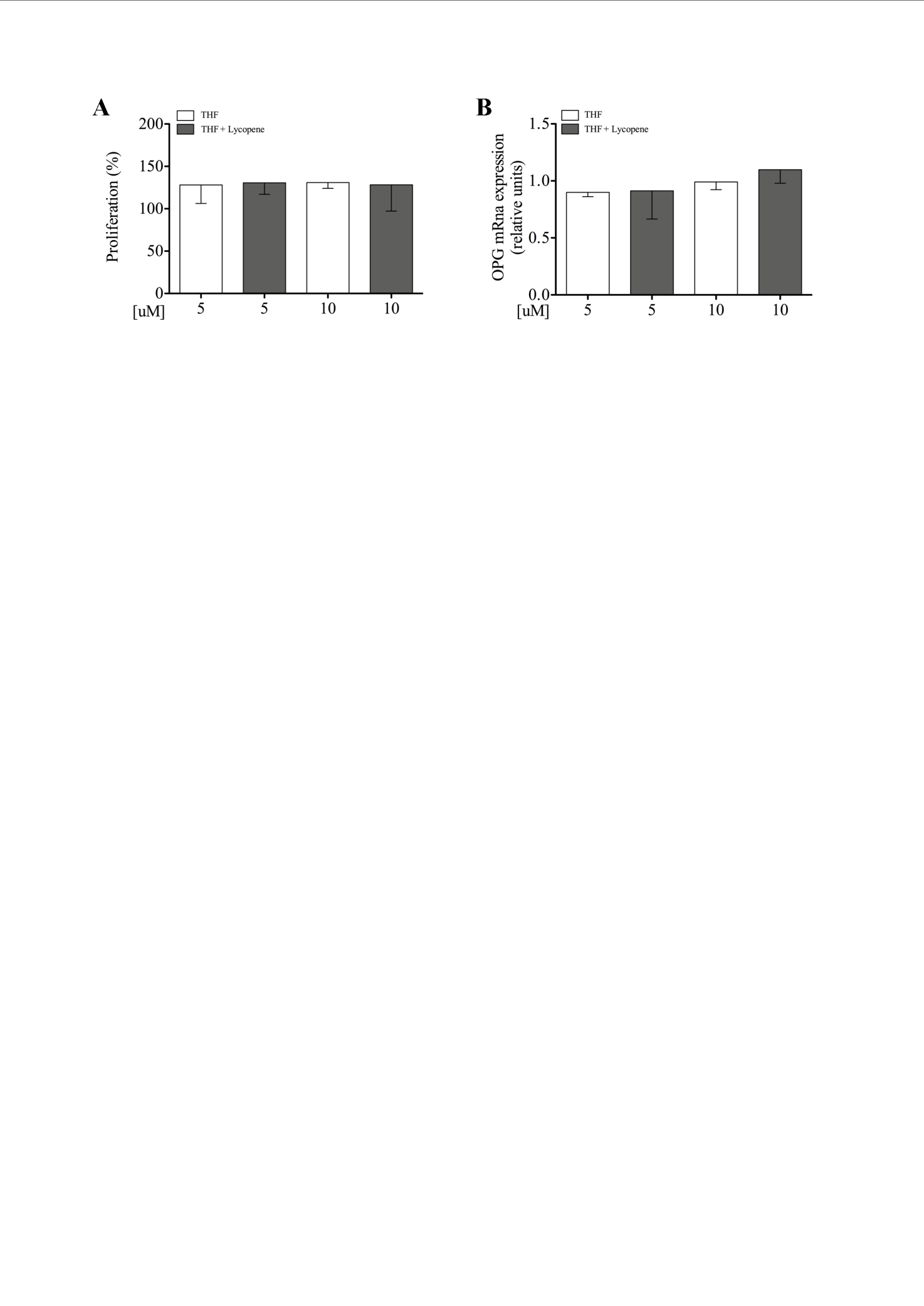

Supplement: Supplementary file 1 — Additional file 1: Table S1. Real-Time primer sequences. Table S2. Baseline demographic and clinical characteristics of participants according to intervention. [file 12967_2020_2238_MOESM1_ESM.docx]
